# Traces of calcium oxalate biomineralization in fossil leaves from late Oligocene maar deposits from Germany

**DOI:** 10.1038/s41598-022-20144-4

**Published:** 2022-09-24

**Authors:** Mahdieh Malekhosseini, Hans-Jürgen Ensikat, Victoria E. McCoy, Torsten Wappler, Maximilian Weigend, Lutz Kunzmann, Jes Rust

**Affiliations:** 1 Palaeontology Section, Institute of Geosciences, Rheinische Friedrich-Wilhelms Universität Bonn, Nussalle 8, 53115 Bonn, Germany; 2 Nees-Institut für Biodiversität der Pflanzen der Universität Bonn, Meckenheimer Allee 170, 53115 Bonn, Germany; 3 Department of Geosciences, University of Wisconsin-Milwaukee, 3209 N. Maryland Avenue, Milwaukee, WI 53211 USA; 4 Hessisches Landesmuseum Darmstadt, Friedensplatz 1, 64283 Darmstadt, Germany; 5 Senckenberg Naturhistorische Sammlungen Dresden, Königsbrücker Landstraße 159, 01109 Dresden, Germany

**Keywords:** Palaeontology, Evolution, Element cycles

## Abstract

Calcium oxalate (CaOx) is one of the most common bio-mineral in extant plants and is believed to serve a variety of functions such as calcium storage and herbivore defense. However, traces of CaOx crystals have rarely been identified in fossil plants, and they are primarily known from fossil gymnosperms, where empty cavities of former CaOx crystals or ghost crystals have been reported from leaf cuticles of some Late Cretaceous and Cenozoic conifers. Here we investigate fossil angiosperm leaves from the late Oligocene Rott Fossil Lagerstätte and report ghost crystals of various shapes, sizes and topology (distribution patterns), and cavities. These micromorphological structures of fossil leaves are compared to CaOx deposits in leaves of extant plants: globular structures in fossil leaves resemble CaOx druses (crystal aggregates) in fresh leaves in size and distribution; and angular or brick-shaped structures in the vascular system of fossil leaves closely resemble prismatic CaOx crystals in the vascular system of extant leaves in both size and topology. Chemically, CaOx druses have survived fossilization as cavities only, and were replaced by organic matter and ghost minerals containing Ca, Si, Al, S, and Fe. The identification of former CaOx remains in leaf fossils provides novel insights on the fate of plant bio-minerals during fossilization. More importantly, it provides an additional aspect of the ecophysiology of fossil plants thus improving the accuracy of palaeoecological reconstructions and can provide a broader perspective on the evolution of CaOx and their rule in plant ecology across geological timescales. Alternative interpretations of the fossil microstructures are discussed but ruled out.

## Introduction

Fossil leaves are an important source of palaeontological information and provide both evolutionary and palaeoecological insights^1–3^. Interpretation of fossil leaves can be relatively straightforward based on broad morphological features such as size, shape, leaf margin, and details of leaf veins and cuticle micromorphology, but often, fossil leaf assignments are tentative and may change over time. Additional diagnostic characters would therefore be highly welcome to support or refute fossil identifications. One neglected feature common in plant fossils are granular structures observed on leaf fossils, which have not yet received a satisfactory explanation. In the well-preserved leaf fossils from the Rott Fossil Lagerstätte (North Rhine-Westfalia, Germany) and some related fossil sites from the Oligocene of that region, numerous granular structures are found, which have been variously explained by previous authors as algal colonies, pollen grains, trichome bases, or papillose structures of the leaf epidermis^4,5^. Winterscheid and Kvaček^6,7^ and Moers^4^ interpreted granular structures on fossil leaves as traces of various algae such as *Botryococcus*, *Tetraedron*, and Chrysophyceae, analogous to similar observations in leaf fossils from the Messel fossil site. Krassilov et al.^8^ in their study of ‘Late Cretaceous Flora of Southern Negev’ present numerous detailed images of fossil leaves which show patterns of granular structures, but these are not discussed in the publication. Generally, no convincing explanation for these rather common granular structures on leaf fossils has been proposed in the literature until now.

**Figure 1:**
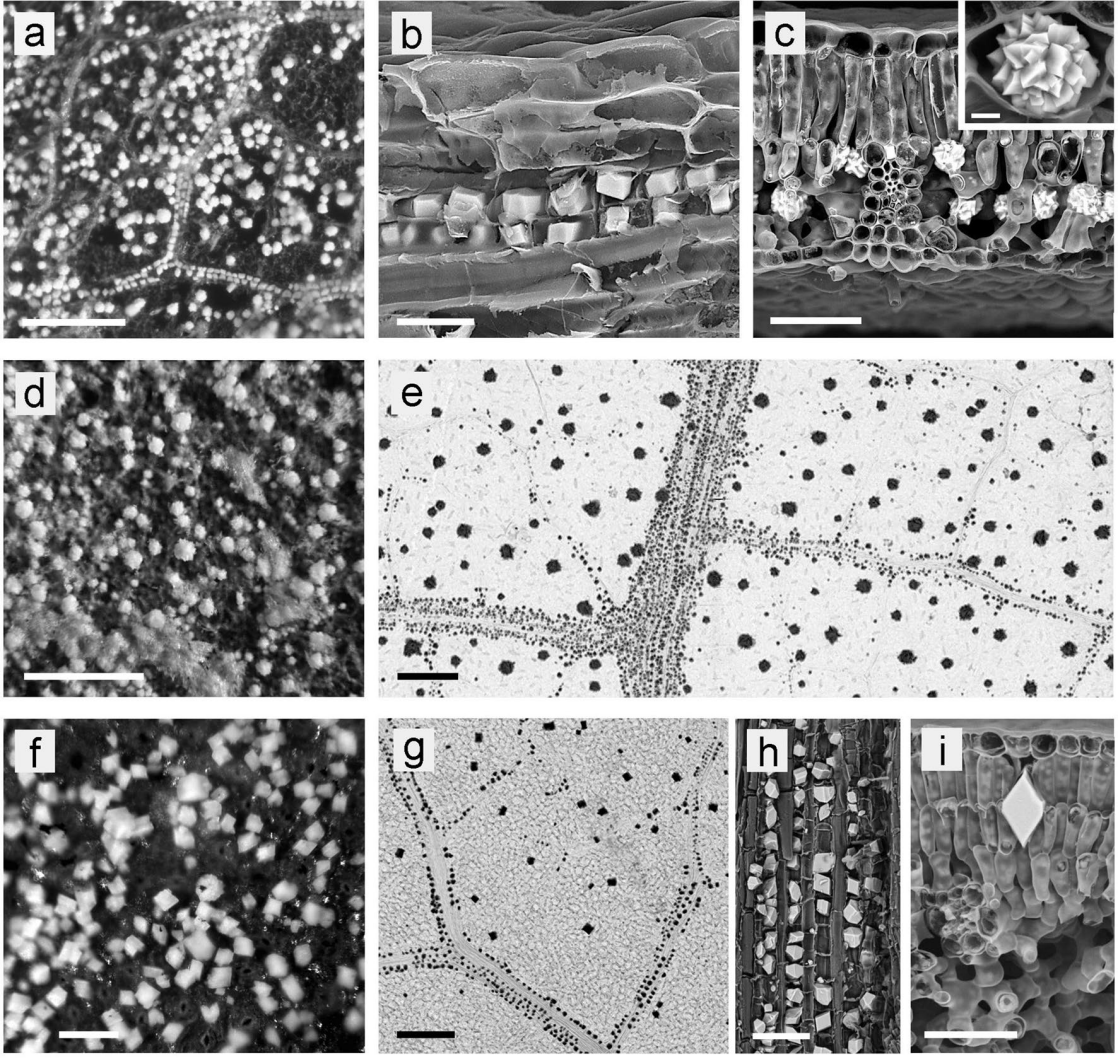
CaOx crystals and druses in leaves of extant plants. (**a**,**d**,**f**) LM images of the ash of carefully incinerated leaves show their distribution in a planar view. (**b**,**c**,**h**,**i**) SEM images of freeze-fractured leaves show crystal and druse morphology in detail. (**e**,**g**) Micro-CT images of CP-dried leaves. (**a**–**c**) *Quercus robur*; high density of druses (15–25 µm) in the areoles and prismatic crystals along the veins. (**d**) *Hedera helix*; leaf densely filled with druses of varying size. (**e**) *Juglans regia*; large druses (50–70 µm) in areoles, and small druses along veins. (**f**) *Prunus laurocerasus*; high density of prismatic crystals (20–30 µm) everywhere. (**g**–**i**) *Parrotia persica;* numerous small crystals along veins and larger crystals in areoles. Scale bars: (**a**,**d**,**e**,**g**) = 200 µm; (**b**) = 20 µm; (**c**,**h**,**i**) = 50 µm; (**f**) = 100 µm; inset in (**c**) = 5 µm.

Many extant plants contain biominerals in various forms, such as mineralized cell walls or mineral particles embedded in tissues^9,10^. The most common forms of plant biominerals are silica bodies (phytoliths)^11^, calcium carbonate (cystoliths)^12^ and various forms of calcium oxalate: individual crystals, druses (crystal aggregates), or raphide bundles^13,14^. Silica biominerals—phytoliths—have been widely studied^15^ and often survive fossilization independent of the surrounding plant tissue, making up microfossil assemblages in their own right^16^. Phytolith analysis therefore has important applications in evolutionary and—especially—archaeological and palaeoecological studies^16–22^. However, the phytolith fossil record is strongly biased towards grasses^16,19^. Calcium carbonate and CaOx are extremely widespread in the plant kingdom and CaOx especially may be found in almost any plant organ or tissue, often specifically in plant groups where silica biomineralization plays a minor role^23–25^. Figure 1 shows a few examples of the variability of the distribution patterns of CaOx druses and crystals in leaves of extant plants, such as *Quercus robur* and *Juglans regia*.

The rich fossil record of silica phytoliths contrasts starkly with the very poor fossil record of calcium biominerals. Despite their ubiquity and importance in extant plants calcium biominerals have rarely been reported from the fossil record^26–29^. This is likely due to the limited chemical stability of both calcium-based biominerals. Calcium carbonate (e.g., cystoliths) is soluble even in the weakest acids, including CO_2_-saturated water, and is therefore unlikely to survive fossilization^30,31^. CaOx itself is less soluble^32,33^, but may be gradually oxidized to calcium carbonate during fossilization, which is subsequently lost from the fossil record. Even if calcium biominerals survive fossilization itself, they are likely to be dissolved during fossilization, which is designed for the extraction of the much more robust silica phytoliths^29^.

Calcium biominerals themselves are thus usually not preserved in the fossil record, but casts (“crystal cavities”) have long been known from the cuticles of fossil conifer leaves^34^. These casts are interpreted as impressions of CaOx crystals in the leaf epidermis as known from extant conifers^35^. These crystal cavities are also used as a minor diagnostic character for conifer taxa such as *Doliostrobus* and *Quasisequoia*^36,37^. Such crystal cavities have been reported from fossils from the Late Cretaceous (e.g., *Quasiseqoia florinii*)^36^, Oligocene (*Glyptostrobus europaeus*)^6^, Paleogene (e.g., *Doliostrobus taxiformis*)^37,38^ and Neogene (e.g., *Cupressospermum saxonicum*)^39^. The occurrence, distribution (adaxial and abaxial leaf surfaces as well as in the mesophyll) and abundance of crystal cavities varies within fossil-species. It has been proposed that this variability in fossils of *Doliostrobus taxiformis* from the Eocene and Oligocene in Europe can be attributed to palaeoecological factors such as habitat and climate^36,37^, but no conclusive evidence has been presented for this assumption. Despite the wealth of angiosperm fossils and the prevalence of bio-minerals in their extant representatives, we are not aware of any reports of “crystal cavities” in angiosperm fossils. We thus observe an odd contrast between calcium biominerals as a common feature of extant angiosperms and the lack of any evidence for these biominerals in the fossil record. On the other hand, there are reports of leaf fossils in which obscure granular structures abound. The present study addresses the question of whether these granular structures correspond to calcium-based biominerals (CaOx crystals and druses, and calcium carbonate grains) or most likely to ghost crystals following the calcium-based biominerals in extant taxa. According to^40^ [page 761] the term crystal ghosts is original defined as a globular assembly of numerous needle-shaped mineral crystals that are organic. In the case presented in the current article, we have CaOx crystals or druses, which left a crystal cavity after they disappeared (e.g. by dissolution) and then were refilled by sediments or organic or mineral crystals (the ghosts).

We therefore re-examine fossil angiosperm leaves from Rott for a better characterization and convincing interpretation of the granular structures. The Rott fossil site is located near Bonn, south of Hennef (Sieg) in the Rhein-Sieg Kreis, North-Rhine-Westfalia, Germany. It is a limnic sedimentary deposit from a freshwater maar lake, famous for its diverse and exceptionally well-preserved plant and animal fossils in the leafy coal beds, diatomite and silica slates of the Rott Formation^5,41,42^. Therefore, it is acknowledged as a fossil lagerstätte^42^. The Rott Formation is dated to Mammal Paleogene zone MP30, which is assigned to the Chattian, uppermost Oligocene (appr. 23 to 24 ma)^4^.

In order to elucidate the identity of the granular structures on fossil leaves, we investigate the fine-scale patterns on fossil leaves and compare them to patterns of CaOx biomineralization of extant plant taxa. Scanning electron microscopy (SEM) and energy dispersive X-ray (EDX) element analyses are used to investigate details of fossil and extant plant materials. Our study specifically aims at answering the following questions:

(1) Do these granules in fossil leaves correspond in shape and location to CaOx druses in modern leaves? (2) Can alternative explanations for the granular structures, e.g., imprints and/or casts of pollen, peltate trichomes, trichome bases, or stomata, be ruled out? (3) Which chemical and biochemical processes affected the leaves containing CaOx during the fossilization? (4) What micromorphological changes happened during fossilization?

## Materials and methods

In the current study, 1120 fossil leaf specimens of the Rott fossil site were examined with a stereomicroscope. All samples are from the collection of the late Heinrich Winterscheid, which is kept in the Goldfuß Museum in the Institute of Geosciences, University of Bonn, Germany. The taxonomic assignments of the fossils derive from the works of H. Weyland between 1934 and 1948^4^. Obvious granular structures were visible on the surface of 64 specimens, which were subject of further detailed examinations. In addition to the partially damaged and contaminated specimen surfaces, we examined freshly split charcoal samples, which could be separated from the fossil block with adhesive tape.

Fresh leaf samples from extant species were collected from the Bonn University Botanic Gardens, Germany. The following species appear in this study: *Carya ovata* (accession 14964, Herbarium T. Jossberger 2406); *Ginkgo biloba* (accession 1894, T. Jossberger 183); *Hedera helix* (accession 8757); *Juglans regia ssp. regia* (accession 9662, T. Jossberger 2418); *Nelumbo nucifera* ssp*. nucifera* (accession 1074, T. Jossberger 2155); *Nymphaea lotus* (accession 41078); *Parrotia persica* (accession 12241, T. Jossberger 534); *Prunus laurocerasus* (accession 34457); *Quercus robur* (accession 1887); *Quercus variabilis* (accession 35458); *Salix miyabeana* (accession 35019, T. Jossberger 2474); *Sideroxylon reclinatum* (accession 34392). Fully developed late-season leaves from adult trees, shrubs and a few aquatic plants were collected in summer and autumn, when CaOx deposits are fully formed (S1). The selection included species or genera closely related to those identified in fossils with granular structures, and additionally some randomly selected deciduous woody species. In total, leaves of more than 50 living species were examined (see Supplementary Table online).

### Microscopy

A stereomicroscope Leica MZ125 (Leica Microsystems, Wetzlar, Germany) was used for selection of samples and examination at low magnification. Detailed light microscopy (LM) was performed with a standard light microscope (Müller optronic, Erfurt, Germany) with large sample stage. Long distance objectives enabled flexible surface illumination with a LED light source. Both microscopes were used with a Swift SC1803 microscope camera (Swift Optical Instruments, Schertz, Texas, US) with 18-megapixel resolution. A Lumix DMC-G70 photo-camera (Panasonic Corporation, Osaka, Japan) with Lumix macro-objective was used for close-up images.

Scanning electron microscopy (SEM) was performed with a LEO 1450 SEM (Cambridge Instruments, Cambridge, UK), equipped with secondary electron (SE) and backscattered electron (BSE) detectors and an EDX element analysis system with Link ISIS software (www.oxford-instruments.com). X-ray images and micro-computer tomography (µ-CT) scans of dry leaves were obtained with a SkyScan 1272 Micro-CT system (Bruker microCT, Kontich, Belgium) in the Institute of Evolutionary Biology and Ecology at the University of Bonn. The images were recorded with a detector of 4032 × 3280 pixels with a pixel size of 1 µm. Visualisation of the µ-CT data was performed with ImageJ-Fiji software (https://imagej.net/software/fiji/).

### Specimen preparation

Fossil samples were cleaned to remove dust, if necessary, with an air-blower or through careful rinsing with distilled water. For SEM examination, small representative pieces were selected, mounted on a sample holder, and sputter-coated with a thin layer (10–15 nm) of palladium. Palladium, in contrast to gold, does not disturb the EDX analyses of relevant elements such as silicon and sulphur, and this thin layer is sufficiently transparent for high-energetic electrons necessary for compositional-contrast BSE imaging.

Fresh leaves were examined to investigate the total amount and distribution of CaOx druses and crystals in a variety of species. Most leaves are not transparent enough to visualize the crystals directly and need to be subjected to a clearing procedure. We found a very simple procedure particularly useful: pieces of the leaves were simply burnt until the organic matter was largely oxidized, reducing the leaf to a brittle piece of ash. For this purpose, fresh or dry leaves were incinerated in a temperature-controlled oven (Brennofen Uhlig U15, Efco GmbH, Rohrbach, Germany) at 600–650 °C. The samples turned white after 5–10 min. In many cases, simple burning over a gas burner was also successful and much faster. Usually, the CaOx structures could be easily visualized directly in the ashes using the stereomicroscope or standard LM. If the ash remnants from the epidermal layers were too thick and not transparent enough, we separated the upper and lower halves of the burnt leaf with transparent adhesive tape such as Tesafilm (Tesa SE, Norderstedt, Germany) and attached each half to a glass slide. Observing the inner side of each half showed the majority of the druses and crystals.

Standard preparation of fresh leaves for SEM and µ-CT: Pieces of fresh leaves were fixed in 70% v/v ethanol + 4% v/v formaldehyde in water for at least 20 h and dehydrated with ethanol. For freeze-fracturing, ethanol-infiltrated samples were immersed in liquid nitrogen and broken randomly. After unfreezing, all samples were critical-point dried (CPD 020, Balzers Union, Liechtenstein) and mounted on sample holders for SEM or µ-CT.

### Plant collection statements

All plant samples collected in this study were taken from species cultivated in the Botanical Garden, University of Bonn. This sample collection complies with relevant institutional, national, and international guidelines and legislation.

## Results

### Fossil leaves

**Figure 2:**
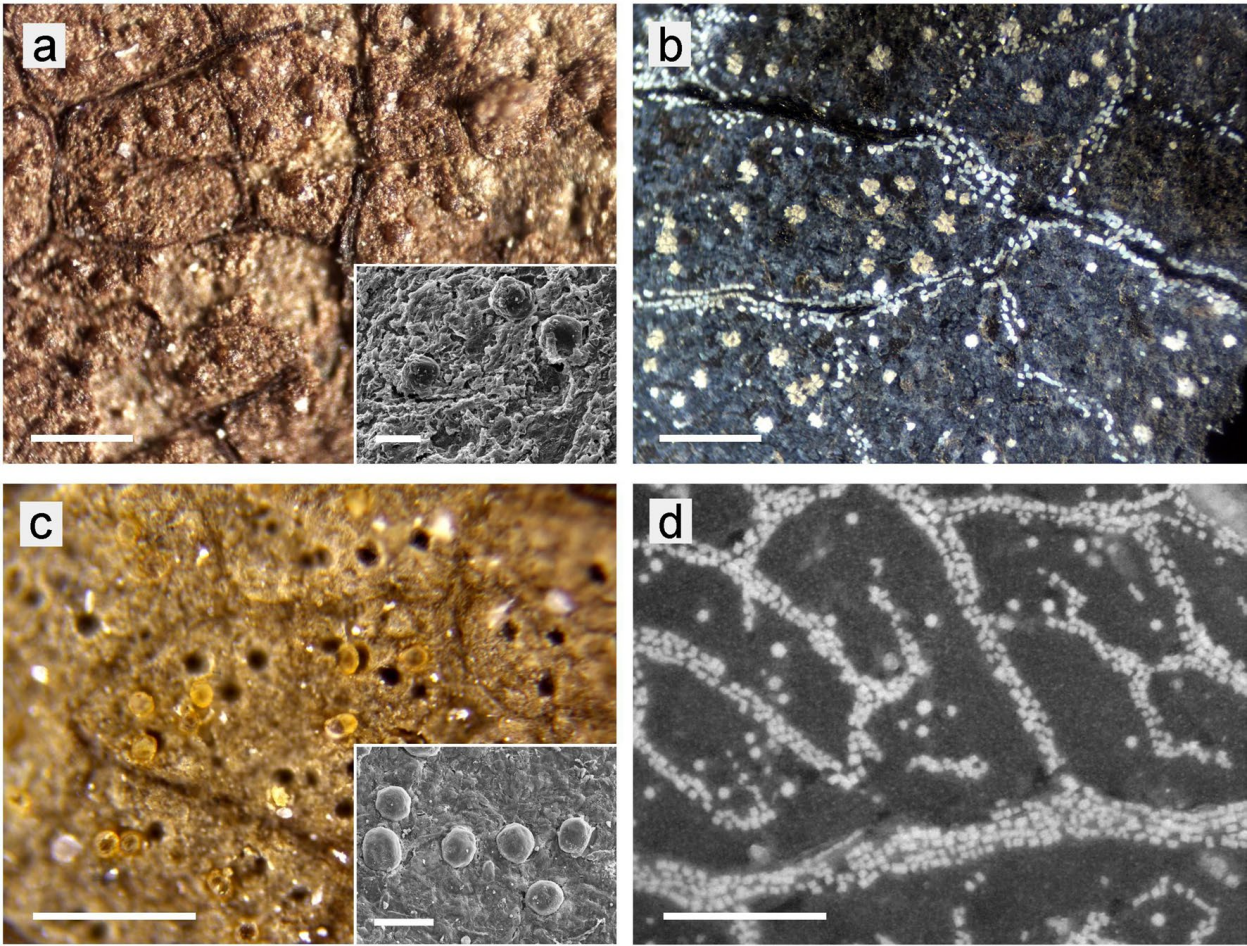
Globular structures in fossil leaves in comparison with CaOx druses of fresh leaves. LM images; surface illumination, 10 × objective. (**a**) Fossil sample Ro-90_1 (*Quercus neriifolia*) with large brown globules; the inserted SEM image shows globules in detail. (**b**) druses of various size and small crystals in a burnt leaf of *Quercus variabilis*. (**c**) Fossil sample Ro-100_5 (*Salix longa*) with yellow transparent globules and many empty cavities, which remained when globules were pulled out during splitting the fossil. (**d**) Druses and crystals in a burnt leaf of *Salix miyabeana*. Scale bars: (**a**–**d**) = 200 µm; insets (**a**,**c**) = 40 µm.

**Figure 3:**
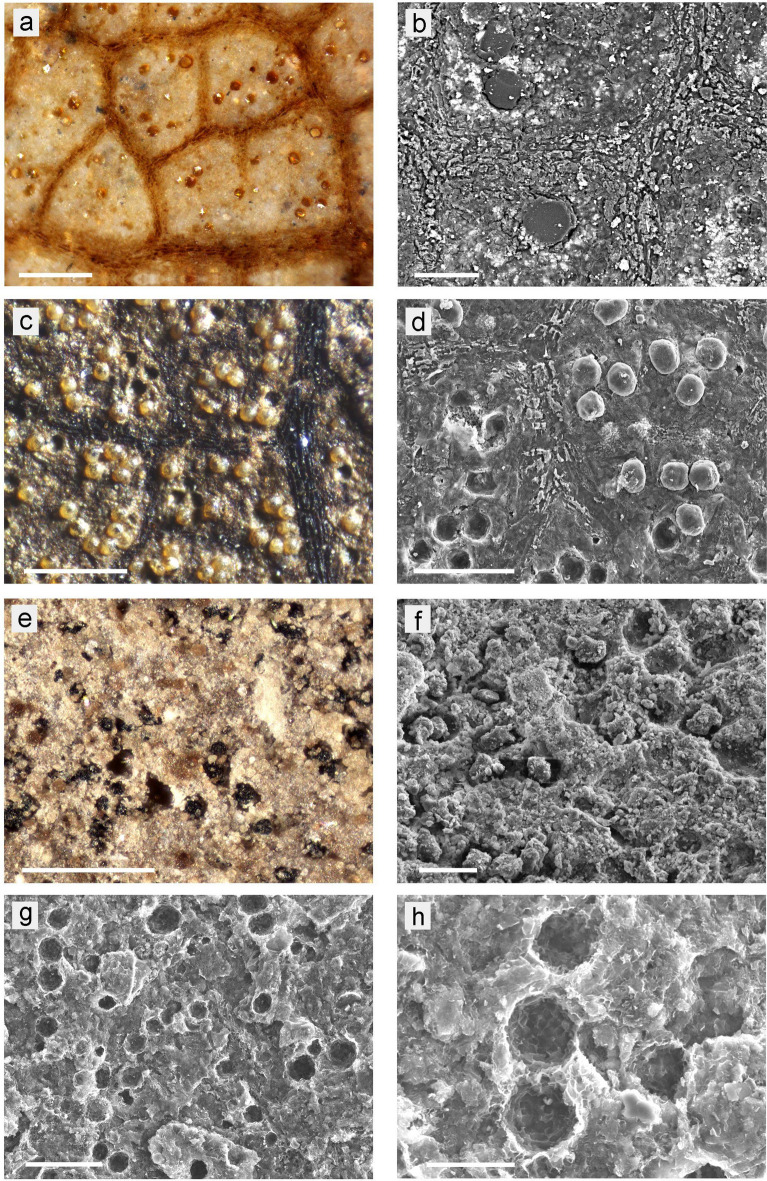
Granular structures in various leaf fossil samples. (**a**,**c**,**e**) LM images; surface illumination, 10 × objective; (**b**,**d**,**f**,**g**,**h**) SEM images. (**a**,**b**) Sample ‘Ro-2.2’ (‘*Sideroxylon salicites’*); the LM image (**a**) shows leaf veins and yellow to brown globules of varying sizes embedded in a bright mineral sediment matrix; in the compositional contrast (BSE) SEM image (**b**), the fragmented globules appear dark, indicating organic material; minerals appear bright. (**c**,**d**) Sample ‘Ro-110.6’ (‘Magnoliopsida’); globules (not fragmented) and holes indicate weak adhesion to the surrounding sediment. Brick-like structures in leaf veins resemble crystals in fresh leaves. (**e,f**) Sample ‘Ro-13.3’ (‘*Nymphaea nymphaeoides*’); LM image shows black serrated particles and (difficult to see) smaller yellow globules. The SEM images shows empty space around granules, perhaps a result of shrinkage. (**g**,**h**) Freshly cleaved area of the leaf coal sample Ro-59.9; numerous empty cavities of various size, up to 30 µm, are distributed evenly. The detail image (**h**) illustrates the angular shape of the cavities which resemble casts of CaOx druses. Scale bars: (**a**,**c**,**e**) = 200 µm; (**b**,**f**,**g**) = 50 µm; (**d**) = 100 µm; (**h**) = 20 µm.

A first examination with a stereomicroscope showed at least 64 samples with obvious granular structures in a total of 1,120 fossil leaf specimens. (Figs. 2a,c, 3a–f). The structures appeared as globular or lobed particles with maximum diameters of 25–70 µm. Most striking were globules, spherical structures of yellow or brown colour with a smooth surface. Several samples contained black structures of irregular or angular shape. Many more samples may have had such granular structures, but they were not sufficiently well preserved for further investigation, or the granules were too small for reliable identification. Some examples of poorly preserved fossils, where recognition of the granules is difficult, are presented in Supplementary Figure S1 online.

Our fossil samples were mostly from diatomite (German term ‚Polierschiefer‘), a bright material rich in silica skeletons of algae, or laminated bituminous shale (leafy coal bed)^42^, a dark brown organic material. Many leaf remnants were very thin as most of the organic material has been lost during fossilization; others consisted of thicker dark-brown layers being coalified remains called compressions^42^. While the old surfaces of the fossils were severely contaminated and damaged, freshly cleaved planes of some leaf coal samples showed their structures in detail. Some specimens showed a distinct pattern of empty cavities with diameters up to 50 µm, which resembled the distribution of CaOx druses in fresh leaves (Table 1). Even small cavities of less than 10 µm were clearly visible.

**Table 1:** List of selected fossil samples with granular structures (traces of CaOx druses).

Fossil sample	Selected fossil samples with traces of CaOx druses				
	Assignment	Type of traces	Size (µm)	Composition	References
Ro-90.1	*Quercus neriifolia*	Globules, brown	40–50	Organic	Figure 2
Ro-100.5	*Salix longa*	Globules, yellow	30–35	Organic	Figure 2
Ro-2.2	*Sideroxylon salicites*	Globules, brown	30–40	Organic	Figures 3, 4
Ro-110.6	*Magnoliopsida*	Globules, yellow	ca. 30	Organic	Figures 3, 4
Ro-13.3	*Nymphaea nymphaeoides*	Globules, yellow	20–25	Organic	Figures 3, 5
		Granules, black	45–55	Mineral + org	
Ro-59.9	*n.n*	Empty cavities	15–25		Figures 3, 6
Ro-58.5	*Zizyphus zizyphoides*	Granules, black	40–50	Mineral	Figure 7
		Globules, yellow	90–100	Organic	
Ro-4.4	*Acer integrilobum*	Globules (pollen?)	ca. 60		Figure 7
Ro-2.2	(2nd leaf fragment)	Angular particles, black	12–20	Mineral	Figure 7
Ro-101.6	*Zizyphus paradisiaca*	Granules, black	75–80	Mineral	Suppl. Fig. S1

### CaOx in fresh leaves

We examined numerous fresh leaves from extant species searching for a correlation of the granular structures in the fossils with structures in living plants. Most of the extant leaves of trees and perennial shrubs contained CaOx druses (crystal aggregates) and individual crystals of varying sizes and distribution densities; Fig. 1 illustrates some of the patterns. Druses have a spherical shape (not elongated) and may be compact with a serrated surface, or with emerging sharp crystal tips. Small individual crystals are often found along veins; solitary crystals in the mesophyll can reach sizes up to 100 µm.

Appropriate methods for determining the density of druses are required for a detailed correlation. Light microscopic (LM) examination of the ash of burnt leaf pieces is particularly useful and allows even small crystals clearly imaged. X-ray imaging and µ-CT are effective techniques for assessing the druses and crystals without any preparation artefacts (Fig. 1e,h).

The size and distribution of the larger druses of various species match the sizes and distribution patterns observed for the fossil granules (see Supplementary Table S1 online). Typically, e.g., in *Quercus* leaves, globular CaOx druses (crystal aggregates) occur preferentially in the areoles whereas CaOx crystals (single or twinned prismatic crystals) are associated with the leaf veins (Fig. 1a–c). Other species contain in areoles and veins only druses (e.g., *Hedera helix*, Fig. 1d; *Juglans regia*, Fig. 1e) or only crystals (*Parrotia persica,* Fig. 1g–i), thus details of the crystal type, size, and morphology are quite variable (Table 2; Supplementary Table S1 online). Quite large druses with diameters of 40–80 µm were found in some *Quercus* species and in Juglandaceae (*Juglans regia, Carya ovata*)—genera which have been reported from the Rott fossil flora—and in *Ginkgo biloba* (Table 2).

**Table 2:** Distribution pattern, size and abundance of calcium oxalate (CaOx) crystals and druses in leaves of extant species presented in this study.

Species	Characterization of CaOx in fresh leaves					
	CaOx in areoles			CaOx along veins		
	Type	Size (µm)	Abundance	Type	Size (µm)	Abundance
*Carya ovata*	Druses	35–50	4	Druses	10–20	5
*Ginkgo biloba*	Large druses	60–100	2	Druses	60–100	4
*Hedera helix*	Druses	25	5	Druses	10	3
*Juglans regia*	Druses	20–55	4	Druses	10	4
*Nelumbo nucifera*	Druses	25–30	3	n. d.		
*Nymphaea lotus*	Small crystals	2–3	2	None		
*Parrotia persica*	Crystals	40–75	2	Crystals	10	3
*Prunus laurocerasus*	Crystals	25–30	5	Crystals	15–20	2
*Quercus robur*	Druses	15–20	5	Crystals	10–15	4
*Quercus variabilis*	Druses (large/small)	45; 20	4	Crystals	15–20	4
*Salix miyabeana*	druses	15–20	3	Crystals	7–10	4
*Sideroxylon reclinatum*	crystals	20–25	3	Crystals	20	2

Abundance of the CaOx bodies is indicated with numbers. 1 = occasional or not clearly apparent; 2 = few; 3 = regular but not many; 4 = many; 5 = densely.

### Morphology of the granular structures in fossil leaves in leafy coal beds and diatomites

Under a binocular microscope, globular particles and venation patterns were clearly visible on many of the selected fossil leaf samples. The sizes of the particles, typically 20–40 µm, match well the sizes of CaOx druses in leaves of extant species, as demonstrated in Fig. 2 with the fossil samples Ro-90_1 (assigned to *Quercus neriifolia*) and Ro-100_5 (*Salix longa*) (Fig. 2a,c) in comparison with leaves from extant *Quercus* and *Salix* species (Fig. 2b,d). However, sizes of up to 100 µm were found for globules in other fossil samples, as well as for CaOx druses of certain extant species (Table 2).

Figure 3 shows various distinctive patterns of granular structures in Rott fossils that are apparent in LM and SEM images. Yellow-to-brown globules in areoles between brown leaf veins (Fig. 3a) are clearly visible contrasting in colour against white background of diatomitic sediment in fossil no. ‘Ro-2.2’ (‘*Sideroxylon salicites*’). Compositional contrast (BSE) SEM image (Fig. 3b) shows fragmented granules embedded in sediment, indicating they differ in chemical compositions. Dark appearance of granules in the SEM image indicates they are organic material, whereas background sediment is a mix of organic and mineral (bright) components.

In several leafy coal bed-based samples like ‘Ro-110.6’ (‘Magnoliopsida’), brown globules, which were on brown organic background between dark brown veins, were more difficult to recognise and distinguish from background by LM due to low colour contrast. Lateral illumination made them more easily visible because most of the globules projected above the level of surrounding leaf area. Globules and a corresponding number of cavities were present in cleaved samples. In ‘Ro-110.6’ (Fig. 3c) globules were evenly dispersed across leaf surface, as often observed in fresh leaves. Brick-like structures in veins appear similar to CaOx crystal patterns along the veins of fresh leaves (Fig. 3d). Sizes of the globular structures may be either variable, as in ‘Ro-2.2’ (Fig. 3a), or nearly uniform. Both variable and uniform sizes were also found as common patterns in CaOx druses in fresh plant leaves; e.g., in *Hedera helix*.

Black serrated particles on a mineral background occur on the whole surface of a large leaf on specimen ‘Ro-13.3’ (‘*Nymphaea nymphaeoides*’) (Fig. 3e,f) and also on some other specimens [Ro-106.1 (‘*Nyssa rottensis*’, Ro-58.5 (‘*Zizyphus zizyphoides*’)]. The size and shape of the serrated granules resemble CaOx druses which are found in many fresh leaves. Smaller yellow globules between the black granules are difficult to recognise under the LM due to low contrast and also by SEM due to the heterogeneous structure of the surrounding area.

Freshly cleaved areas of the leafy coal bed sample ‘Ro-59.9’ (Fig. 3g,h) showed numerous empty cavities which resembled CaOx druses of fresh leaves in size and distribution. The polygonal shape corresponds to the angular surface of druses, whereas the smooth surface of the organic globules (Fig. 3c,d) seems to be caused by their shrinkage. The variation in size, up to 30 µm, is remarkable and corresponds to extant druses. On such clean specimens, cavities of less than 10 µm diameter can be clearly recognised.

### Compositional analyses of granules in leaf fossils

**Figure 4:**
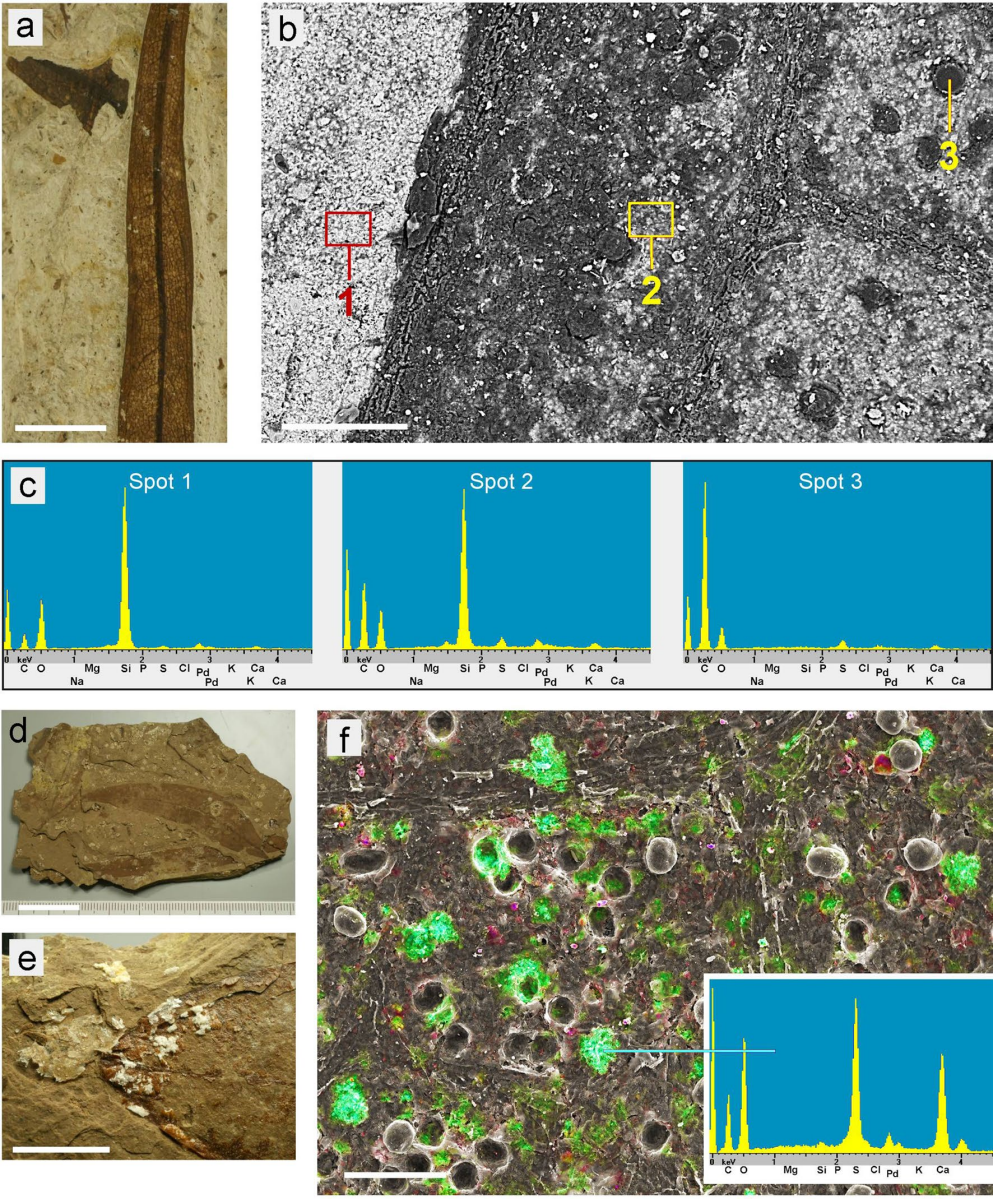
Analyses of samples with globules. (**a**–**c**) ‘Ro-2.2’ (‘*Sideroxylon salicites’*). (**a**) Overview photo of the brown leaf embedded in white mineral. (**b**) Compositional contrast (BSE) SEM image showing organic leaf material dark, background mineral (left part) bright, and ghost mineral inclusions in the leaf area (bright). The fractured globules (e.g., Spot 3) appear dark, indicating organic material. (**c**) EDX spectra show the composition of background sediment (Spot 1; mainly Si and O), the leaf area ‘Spot 2’ (Si, C, O, and traces of S and Al), and globules (Spot 3; mainly C with little O and S). (**d**–**f**) ‘Ro-110.6’ (‘Magnoliopsida’). (**d**,**e**) Overview photos showing the leaf embedded in coal. (**f**) Combined SEM topographic contrast and element-mapping image showing Ca in green and Si in red; organic structures are in grey scales. The EDX spectrum in the box shows the co-localization of Ca, S, and O as calcium sulphate (CaSO_4_). Globules are organic (C and little O). Many cavities contain a shell of CaSO_4_ which had surrounded the globules which have been torn out of the coal matrix. Calcium sulphate occurred also on other parts of the sample in form of gypsum deposits (**e**). Scale bars: (**a**,**e**) = 5 mm; (**b**,**f**) = 100 µm; (**d**) = 20 mm.

**Figure 5:**
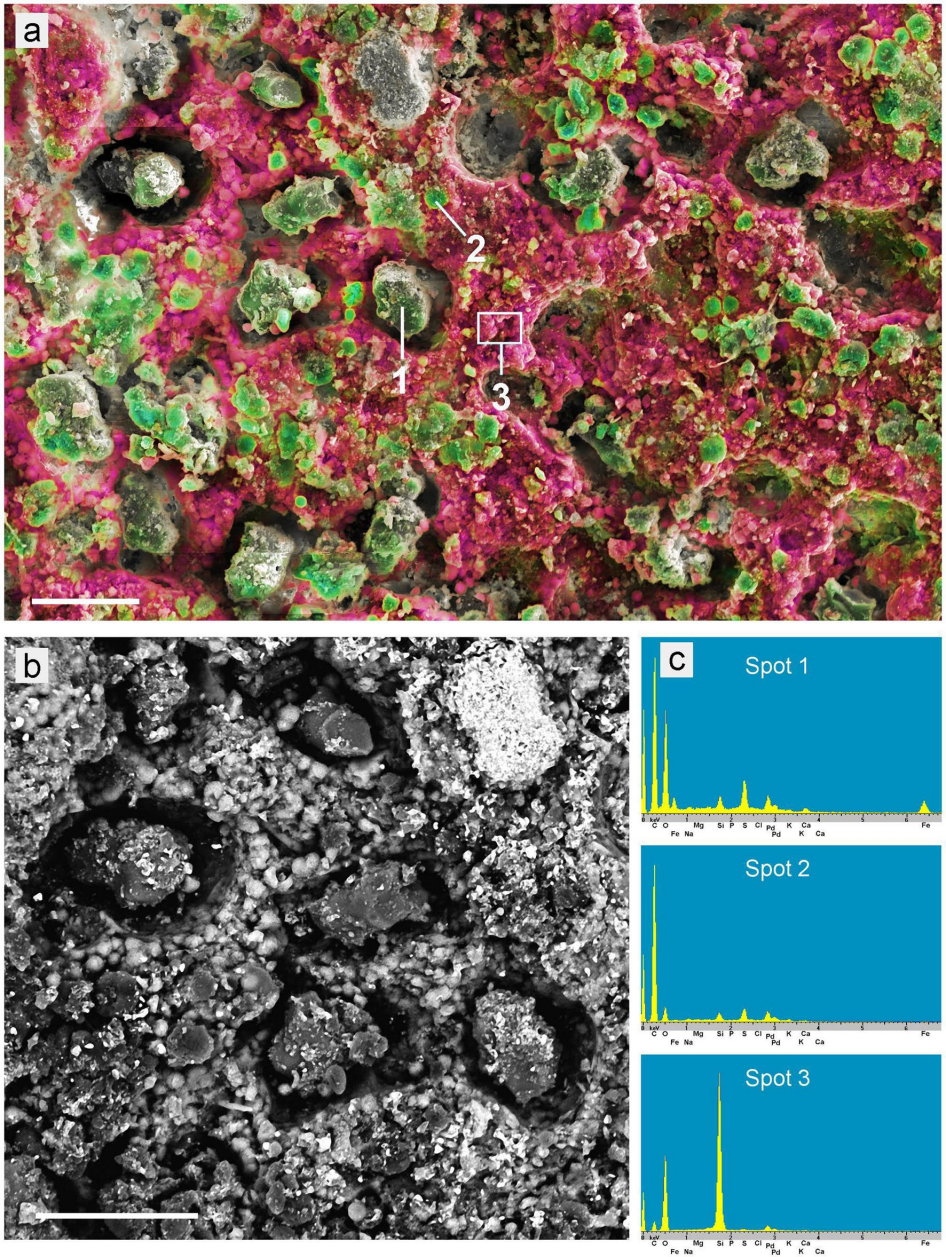
Element distribution in the fossil sample ‘Ro-13.3’ (‘*Nymphaea nymphaeoides’*). (**a**) Combined SEM topographic contrast and element-mapping image showing Si in red and C in green colours. Small globules of organic material (e.g., Spot 2) are difficult to recognise between other organic particles. (**b**) Detailed BSE image of a section of (**a**) illustrating the grainy opal-like structure of the background sediment. (**c**) EDX spectra show the composition of black granules (Spot 1: C, O, Fe, S), small globules (Spot 2: mainly C), and background sediment (Spot 3: Si, O). Scale bars: (**a**,**b**) = 40 µm.

EDX element analyses of selected fossil samples were conducted with an SEM (Figs. 4, 5). In the mineral-based sample ‘Ro-2.2’ (‘*Sideroxylon salicites’*) (Fig. 4a–c) the globules appear dark in the BSE image (Spot 1), whereas mineral ghost inclusions in the leaf and the mineral background appear bright. EDX spectra show that the globules are organic and contain mainly carbon (C) with a little oxygen (O) and sulphur (S). The mineral background consists of silica (SiO_2_). Calcium was not found.

The globules in the brown-coal based sample ‘Ro-110.6’ (‘Magnoliopsida’) (Fig. 4d–f) consisted of organic material, composed mainly of C with a little O and S, but they were accompanied by calcium sulphate (Ca, S, O), which surrounded many of the organic globules like a shell with a serrated surface. Small amounts of silicon (Si) and iron (Fe) occurred in the background brown-coal material.

The mineral sample ‘Ro-13.3’ (‘*Nymphaea nymphaeoides’*) (Figs. 3e–f, 5) had serrated black granules, marginally smaller than the corresponding cavities in the sediment, and smaller yellow-to-brown spherical globules. The red colour in Fig. 5a indicates a high Si content in the surrounding sediment; the BSE image (Fig. 5b) illustrates the opal-like structure consisting of fine spherical silica particles. EDX spectra (Fig. 5c) revealed that the small globules (Spot 2) were organic material (mainly composed of C), the black granules (Spot 1) contained mainly C, O, Fe, and S, and the mineral background was silica (Si, O). The small spherical organic globules are difficult to recognize between other organic structures, but they are also visible in LM as distinct structures (see Fig. 3e).

Other samples: Globules with a size of 30–70 µm were the most obvious type and relatively abundant. Some samples had much smaller globules with less characteristic shape (e.g., Ro-110.27), but their size and density are in accordance with similar patterns of CaOx druses in certain fresh leaves such as *Prunus laurocerasus, Sideroxylon reclinatum* (Table 2).

The analyses demonstrate that the granular structures in the leaf fossils do not currently consist of calcium oxalate. This is the result of the decomposition of the original biomineral, leaving behind cavities which subsequently filled with organic or inorganic material. These ghost minerals roughly replicating the shape of original biomineral. Spherical globules were found to be purely organic, whereas serrated globules contained minerals; Fe and S were usually found in black particles.

## Discussion

**Figure 6:**
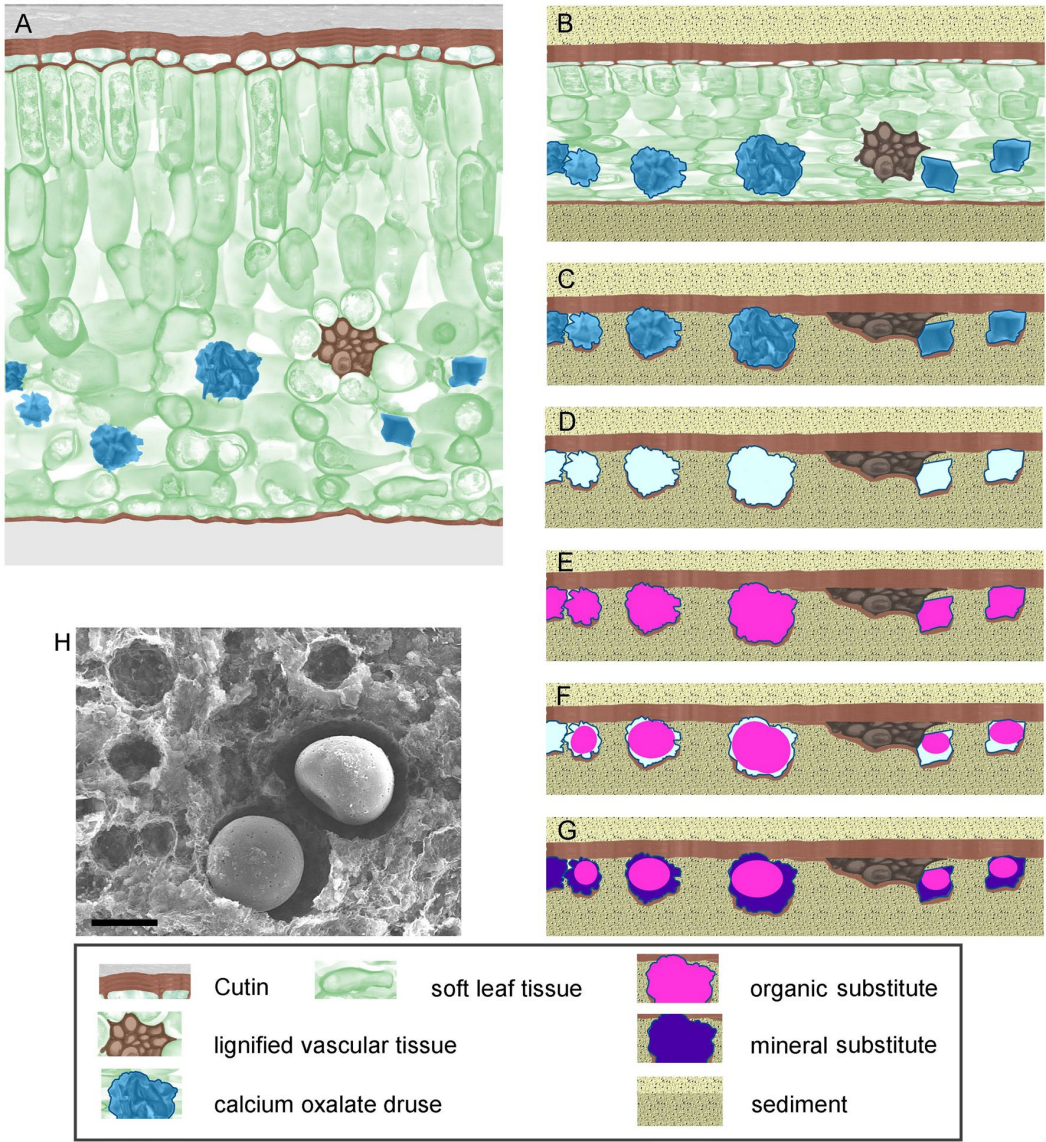
Model of the fossilization processes that lead to the formation of globular and serrate replications of CaOx crystals and druses. (**A**) Distribution of CaOx and other structures in a fresh leaf. (**B**–**G**) Fossilization steps. (**B**) Covering with sediment; compression, loss of water; (**C**) Decomposition of soft organic tissue material; (**D**) Dissolution of CaOx forms voids; (**E**) Voids are filled with organic matter, perhaps cutin components; (**F**,**G**) partial shrinkage of organic material forms globules due to surface tension; new voids formed by shrinkage are filled with ghost mineral components. (**H**) SEM image of a freshly cleaved plane of leaf coal sample Ro-59.9 (see Fig. 3g–h). Larger cavities contain shrunken remnants of organic material, whereas smaller cavities are empty. Scale bar = 20 µm.

The present study illustrates the distribution, micromorphology and elemental composition of granular structures in fossil leaves and compares them to those of CaOx druses and crystals in extant leaves. We provide a brief review of a possible fossilization scenario of the leaves that could lead to the formation of the brownish granular structures in fossil leaves (Fig. 6). Leaves of terrestrial plants and freshwater plants sank into the anoxic depths of a maar lake and were preserved in different sediments. During fossilization, parts of the organic material decomposed; any remnants are compressed. Vasculature with its lignified cellular walls, cutinized peripheral walls and mineral inclusions is more likely to be preserved. During fossilization, CaOx will eventually decompose or dissolve. If the sediments are already consolidated and sufficiently hardened, then the disappearance of the druses and crystals will leave cavities which—depending on local conditions—will be filled either with organic or inorganic ghost minerals. Deposition of very fine material such as amorphous silica may form replicas which resemble the former CaOx crystals (ghost crystals, see Fig. 5). In an inorganic mineralogical system these replicas would be called pseudomorphs.

The mostly spherical shape of the globules requires an explanation, since CaOx crystals even in druses are usually polygonal and very angular structures. We propose that the spherical shape of the organic globules results from shrinkage of the casts. Organic material, which has filled the voids, is unlikely to be perfectly stable. It may lose material and shrink, and, as it is a highly viscous resin-like material, its surface tension in the wet environment may force it into the spherical shape. The shrinkage also explains why the organic globules easily become detached from the surrounding sediment, leaving spherical cavities at their positions in the separated fossil samples. Further shrinkage and dissolution of the organic inclusions leaves voids which may be finally filled by inorganic ghost minerals from the environment, resulting in the black ferruginous particles or in the calcium sulphate shell observed around some of the organic globules.

Granular structures like the ones here studied have been previously reported from fossil plants and have been variously interpreted as pollen(clumps), algal colonies, trichomes and trichome bases^5^, or ‘subcrustations, preserving epidermal structures’^8^. The study of Krassilov et al.^8^ on the ‘Late Cretaceous Flora of Southern Negev’ includes a wealth of excellent images of fossils; many of them show patterns of granules which resemble the granules here studied, but the authors do not provide an explanation for the structures.

**Figure 7:**
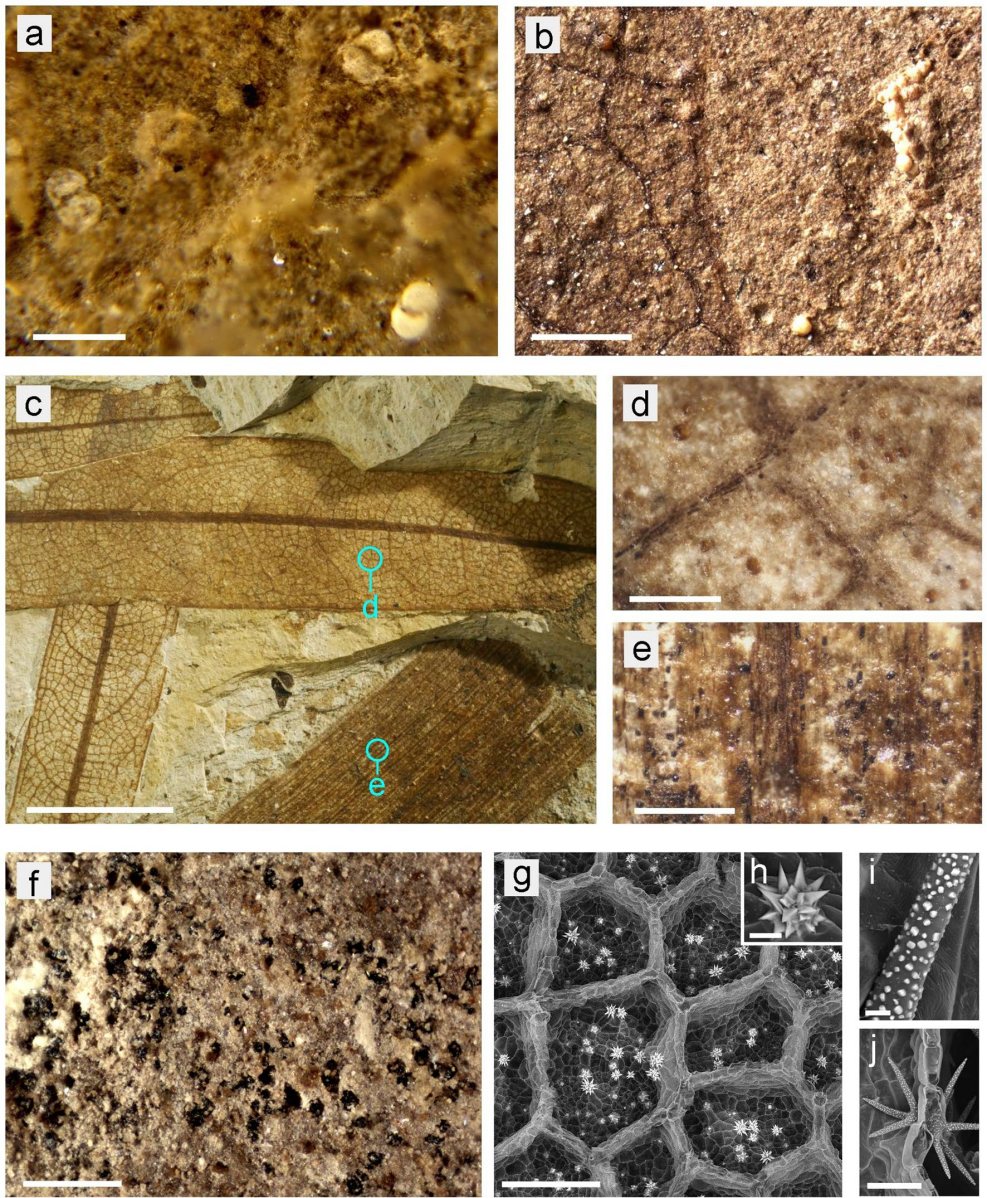
Topics for Discussion: (**a**–**e**) Differentiation between traces of druses and pollen. LM image of Sample Ro-58.5 (‘*Zizyphus zizyphoides*’) with pollen-like structures randomly distributed. Their shape resemble conifer pollen. (b) Sample Ro-4.4 (‘*Acer integrilobum*’); the globules of ca. 100 µm diameter, distributed on (left side) and beside (right side) leaf area may be pollen. (**c**–**e**) Sample Ro-2.2 (‘*Sideroxylon*’) with different leaves. The larger ones contain globules of different size (**d**), the other one small black particles (**e**), no globules were found in the mineral sediment. Such patterns cannot originate from pollen. (**f**–**j**) Uncertainty in the identification of leaf fossils. (**f**) LM image of sample Ro-13.3, which has been assigned to ‘*Nymphaea nymphaeoides*’, shows black granules. (**g**–**h**) SEM images of a fresh leaf of *Nelumbo nucifera* (Lotus) show CaOx druses in similar distribution and size as sample Ro-13.3; inset (**h**) shows a single druse in detail. (**i**,**j**) SEM images of mesophyll cells in a fresh leaf of *Nymphaea lotus*, carrying only small CaOx crystals (< 5 µm) (**i**) on the surface of sclereid cells (**j**). Scale bars: (**a**) = 100 µm; (**b**) = 500 µm; (**c**) = 5 mm; (**d**,**e**,**f**,**g**) = 200 µm; (**h**,**i**) = 10 µm; (**j**) = 100 µm.

The globular structures in our samples were mostly found within the boundaries of the leaf fossils, but occasionally also in the surrounding sediment (Fig. 7a,b). The latter might indicate an extraneous origin—e.g., the presence of pollen—rather than an integral component of the leaf such as CaOx crystals. However, we propose that CaOx druses in decomposing plant remains can be partly dislocated, e.g., by water movement. Druses in leaves occur in different conditions; some are enclosed by massive cell walls whereas others are almost free and only weakly connected to cellular structures (Fig. 7g). The weakly connected druses may easily become dislocated from tissue during decomposition^43^. Thus, their traces might be found outside the leaves in the sediment. Pollen and algae may be found in fossil samples, and the fossilized plants from Rott have been described as ‘rich in pollen’^4,44,45^. A detailed study of the distribution of the granular structures shows that in many of our samples, such as Ro-2.2 (‘*Sideroxylon*’) (Fig. 7c–e), the distribution of granules is highly regular. This indicates that the structures were an integral part of the leaf, since pollen would be expected to be randomly distributed on and around the leaf. Two large leaves of one type in Fig. 7c,d show a similar distribution of globules to each other, whereas a different type of leaf on the same specimen (Fig. 7c,e) has both a different type of fossil particles (smaller, black) and a different distribution. No globules were found in the mineral sediment surrounding the leaves of this specimen, clearly indicating that the granular structures are part of the fossils proper and also that both size and distribution may be characteristic for the two different types of leaf here preserved. Various leaf structures, such as peltate trichomes, trichome bases, or calcium carbonate cystoliths, may also cause visible traces in fossilized leaves. However, extant relative plants of most of the assumed fossil-species from Rott bear very few peltate trichomes on their leaves and no cystoliths. The distribution pattern of trichomes—if present—on leaves differs clearly from the distribution of the granules in fossils: most trichomes are located on the abaxial leaf side on the veins; trichomes on the adaxial side usually occur singly in the areoles between veins, but not in such a high density.

Of course, a careful and critical examination of the fossils is generally necessary to avoid misinterpretations. Some indications are helpful to identify granular structures as traces of former druses: their spherical shape, their occurrence in the remnants of the parenchym, and the regular distribution within the leaf, which resembles that of druses in fresh leaves. In case of the Rott Fossil Lagerstätte it seems to be very unlikely that the cavities filled with granules are originated by soft-tissue remains such as paltate trichomes or pollen clumps. If such structures are preserved as impressions in fine-grained sediments like leafy coal beds any additional micromorphological structures should be visible, e.g., pollen clumps: individual grains of pollen clumps, wall structure and aperture of pollen grains; cell structure, bases and glands of peltate trichomes. None of these features have been recognized by our observations.

Fossilization conditions in the former ‘Rott Lake’ were major factors for the decomposition of the organic plant material and the formation of fine-grained or amorphous inorganic deposits in the sediments including the elements Si, Al, Fe, S, and sulphate ions^4^. Anaerobic conditions, indicated by the presence of pyrite, may have inhibited the oxidation of calcium oxalate to carbonate, thereby facilitating preservation.

The presence of calcium oxalate druses or crystals and their distribution patterns as well as their fossil traces may be utilized as additional useful micromorphological features for the identification of fossil plant taxa in the future, if more records are available and if these records can be unambiguously related to extant taxa. Pattern, shapes and sizes of CaOx traces could be particularly helpful if leaf cuticles are not preserved. Angiosperms show a great variability of biomineralization patterns including CaOx, particularly dicotyledons contain almost all forms of CaOx druses and crystals. Some families of monocotyledons (e.g., Araceae, Arecaceae) contain CaOx raphide bundles, others such as Poaceae (grasses) are usually mineralized with silica but not with CaOx. In Gymnosperms, CaOx occurs in different forms: Conifers usually contain small crystals (< 10 µm) on the surfaces of mesophyll cells, whereas Ginkgo and Cycadeae usually contain druses of 30–80 µm diameter^46^. Sporophytes rarely contain CaOx; small crystals have been found in few fern species^47^.

Unfortunately, there is still a striking scarcity of literature on calcium oxalate crystals in the leaves of extant plant taxa also reported from the fossil record. The absence of detailed data on biomineral occurrence, sizes and topology renders an interpretation of fossil patterns a challenging exercise.

Particularly for the fossil leaves from Rott, the future utilization of CaOx traces in fossil leaves may provide a valuable set of additional characters for the species- or genus-level identification of appropriately preserved leaf material. Identification of fossil leaves is particularly difficult or impossible if only leaf fragments or leaves without cuticles are preserved. Many early taxonomic assignments of leaf fossils from Rott such as the works of H. Weyland between 1934 and 1948 have been shown to be partially unreliable due to poor preservation^5^ and several revisions of have been made in the recent years^6,7,48^.

Our comparisons of fossil samples with extant plants showed inconsistences in several cases. The shapes and distribution patterns of granules in fossil leaves assigned to *Sideroxylon salicites* were different from those of CaOx druses in extant *Sideroxylon* leaves, casting serious doubt on the fossil identification. However, the fossil flora of Rott is currently under taxonomic revision and justification or adjustment of the determination is expected. Similarly, fossil leaf fragments designated as *Nymphaea nymphaeoides* (Nr. Ro-13.3) (Fig. 7f) contained numerous globular inclusions whereas recent *Nymphaea* leaves (Fig. 7i,j) contain only minute CaOx crystals and lack druses. Extant Lotus (*Nelumbo nucifera*, Fig. 7g,h) however, which has similar leaves and ecology, contains druses in similar distribution and size as the globules in the fossil sample named *Nymphaea*. Fossils of both genera of water plant—*Nymphaea* and *Nelumbo*—have been reported from the Rott fossil site^49,50^, but the data in the present study indicate that at least some *Nymphaea*-fossils might be better placed in *Nelumbo*.

The venation patterns of fossil leaves are an important characteristic for identification of species. If our observations that the brick-like structures in the venation largely result from the traces of former CaOx crystals are validated in future studies, it will be an important step to a more precise interpretation of the fossil record of plants. Based on ongoing research, we will be able to add data from other fossil sites of different stratigraphic positions which will demonstrate similar granular structures in fossils of some gymnosperms (e.g., *Ginkgo*, leaves without trichomes) and other dicotyledonous taxa.

In conclusion, the identification of cavities and imprints in fossil leaves as former CaOx crystals and druses has considerable consequences:

- It improves our understanding of micromorphological structures of fossil leaves and the processes taking place during fossilization.
- It may provide a basis for a study of the evolution of plant biomineralization across a range of different lineages.
- It could provide an additional set of characters for improving taxonomic assignments of fossil leaves if appropriately preserved.
- It may become a useful additional aspect of leave trait analyses.

Currently, our interpretation of biominerals in fossil plants is severely limited by our rudimentary knowledge of biomineralization in living plants. A comprehensive database of current biomineralization patterns would be highly desirable to get progress in this topic. It promises to be a valuable tool in palaeobotany and greatly improve our understanding of both plant evolution and palaeoecology.

## Supplementary Information

Supplementary Information.

## Data Availability

The data that support the findings of this study are based on microscopic images which are archived in the Microscopy/SEM facilities of the Institute of Geosciences and the Nees Institute, Bonn. Images are available on request from the corresponding author M. Malekhosseini and from co-author Prof. Dr. M. Weigend.
