# Supplementary material for: Traces of calcium oxalate biomineralization in fossil leaves from late Oligocene maar deposits from Germany
Source: Sci Rep. 2022 Sep 24;12:15959. doi: 10.1038/s41598-022-20144-4 (PMC9509364; doi:10.1038/s41598-022-20144-4)
Supplement: Supplementary file 1 — Supplementary Information. [file 41598_2022_20144_MOESM1_ESM.pdf]

Supplementary Information

Title: Traces of calcium oxalate biomineralization in fossil leaves from late Oligocene maar deposits from Germany.

Mahdieh Malekhosseini, Hans-Jürgen Ensikat, Victoria E. McCoy, Torsten Wappler, Maximilian Weigend, Lutz Kunzmann, and Jes Rust

Supplementary Table S1

Calcium oxalate crystals in fresh leaves, based on LM examinations											
	Crystals at veins			Crystals in areoles, major type			Crystals in areoles, minor type				
	Type	Amount	Size (µm)	Type	Amount	Size (µm)	Type	Amount	Size		
Acer griseum (BG, 36732)	crystals	4	10-12	small druses	2	15	crystals	1	ca. 15		
Acer pseudosieboldianum (BG, 36830)	crystals or aggregates	5	12-15	crystals or druses	1	15					Amount of Crystals:
Acer saccharum (BG, 35481)	crystals	3	ca. 10	0	0						0 = none
Acer sempervirens (BG, 3349)	crystals	5	15-20	crystals	ca 3	15-20					1 = occasional or not sure
Agarista populifolia (BG 35470)	crystals	4	30-45	crystals	3	35-50 µm					2 = few
Berberis jamesiana (BG, 33416)	0	0		0	0						3 = regular; not many
Betula pendula (BG, 25573)	crystals	3	10-12	druses	3	10-15 µm					4 = many
Carpinus botulus (BG, 12474)	0	0		large crystals	3	40					5 = densely
Carpinus kawakami (BG, 34895)	0	0		large crystals	3	60-90	small druses	3			
Carpinus orientalis (BG, 09661)	0	0		large crystals	3	50-65					
Carya ovata (BG, 14964)	crystals	5	15-20	druses (aggr.)	3	50-70					
Carya tomentosa (BG, 1898)	crystals	4	10-15	large druses	4	30-40	small druses	4	10-20		
Castanea spec	druses	3	8-15	druses	4	5-25					
Celtis caucasia (BG, 36534)	crystals	2	10-15	cystoliths	5	70	small crystals	3 (?)	10-12		
Celtis occidentalis (BG, 10137)	crystals	3	10-15	cystoliths	4	50-80	druses	3	10-15		
Fagus grandifolia (BG, 27351)	crystals	3	ca. 25	druses	1	ca. 20	small crystals	1	ca. 10		
Fagus sylvatica (BG, 15397)	crystals	4	12-16	druses	3	15-20					
Ginkgo biloba (BG, 20184)	large druses	5	70-100	large druses	1	70-100					
Hamamelis japonica (BG, 36254)	crystals	4	ca. 20	druses	4	ca. 20	crystals (?)		ca. 20		
Hedera helix (BG, 8757)	druses	1	20-30	druses	5	20-30					
Juglans regia (BG, 36412)	small druses	3	7-12	large druses	4	40-50					
Leitneria floridana(BG, 34403)	0	0		0	0						
Leucosidea sericea (BG, 36706)	druses	4	ca. 15	druses	3	ca. 15					

Magnolia ernestii (BG, MM)	0	0		0	3						
Magnolia grandifolia (BG, MM)	0	0		0	0						
Myrica pensylvania (BG, 3722)	druses	5	12-16	few druses	1	12-16					
Nyssa aquatica (BG, 37827)	crystals	4	10-15	0	0						
Nyssa sylvatica (BG, 12270	crystals	5	10-13	druses	2	15-22					
Ostrya virginiana (BG, 38551)	0	0		crystals	4	25-30					
Parrotia persica (BG)	small crystals	4	15-20	large crystals	2	30-50					
Platanus orientalis (BG, 1970)	crystals	2	15-18	druses (?)	2	10-15					
Platanus racemosa (BG, 37232)	0	0		druses	2	12-18					
Platycarya strobilacea (BG,12057)	0	0		large druses	3	80-100					
Prunus laurocerasus (Ens)	crystals	2	15-25	crystals	4	25-30	druses	2	ca. 35		
Quercus acutissima (BG, 39524)	crystals	5	8-10	druses	4	18-30	small druses	1	ca. 10		
Quercus castaneifolia (BG, 35005)	crystals	3	10-14	druses	3	ca. 25					
Quercus imbricaria (BG, 21883)	crystals	5	8-12	druses	5	10-15	large druses	1	ca. 20		
Quercus laurifolia (BG, 35456)	crystals	3	10-14	druses	3	20-25					
Quercus macrocarpa (BG, 12423)	crystals	4	10-18	druses	5	10-20					
Quercus pagoda (BG, 33305)	crystals	3	6-12	large druses	4	30-40	small druses	3	10-20		
Quercus robur (BG, 30830)	crystals	4	10-20	druses	5	10-20					
Salix helvetica (BG, MM)	crystals	3	8-15	druses	2	ca. 15					
Salix purpurea (BG, 17982)	crystals	3	10-15	druses	1	10-15	crystals	1	10-15		
Sideroxylon reclinatum (BG, 34392)	crystals	4	ca. 20	crystals	2	20-25					

Abundancy and properties of calcium oxalate crystals and druses in fresh leaves of various trees from the Botanical Garden, Bonn

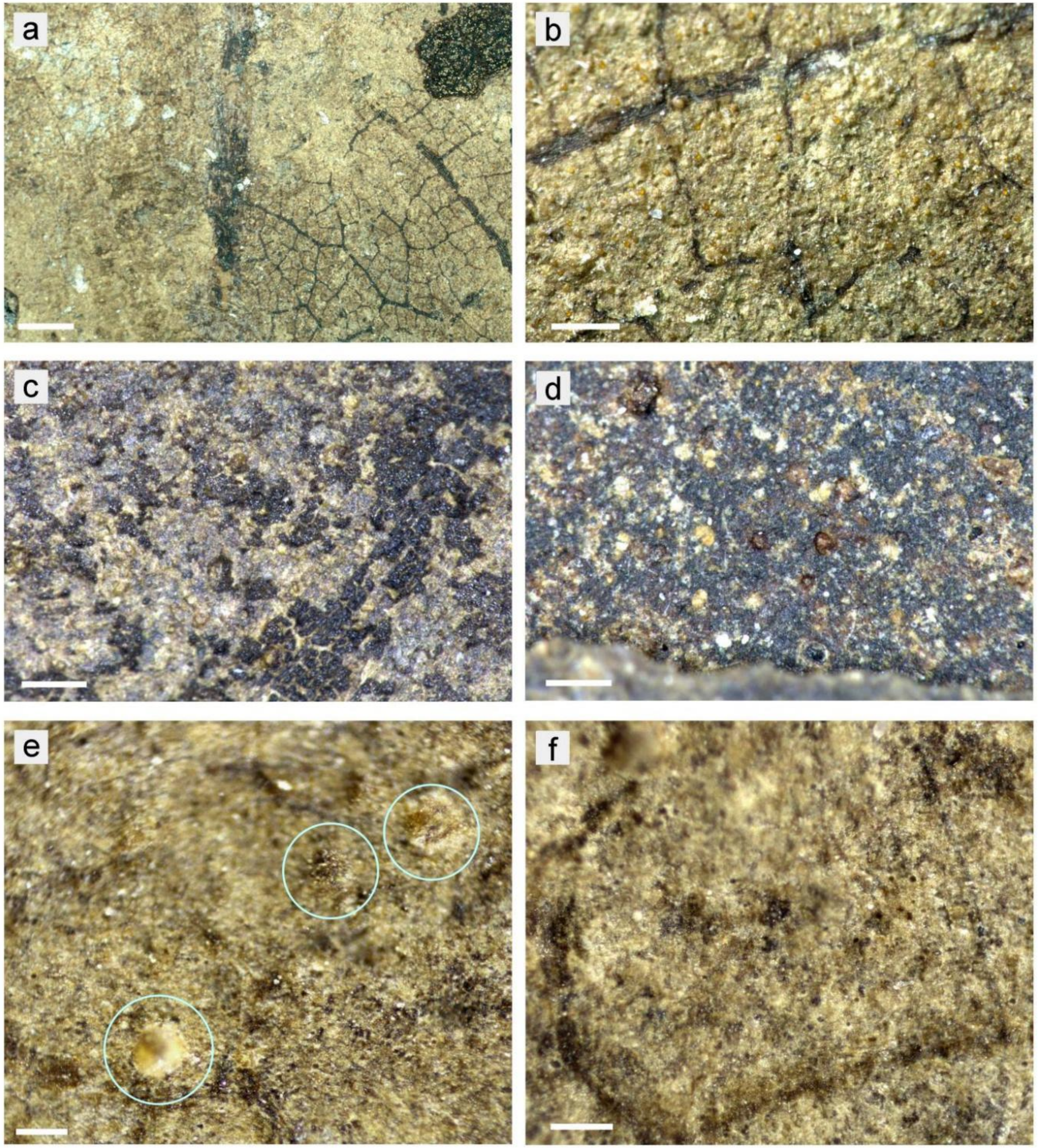
a
b
c
d
e
f

Supplementary Figure S1: Some examples of LM images of fossil samples illustrating difficulties to recognise granules. (a-b) Sample Ro-110 (Magnoliopsida) and (c-d) sample Ro-101.6 (Zizyphus) with fragmented leaf remnants. Granules can be seen in few places only. (e): Sample Ro-58.5 (Zizyphus); granules with a rough surface are more difficult to recognise than smooth ones (both encircled). Their detection requires optimal illumination and stereoscopic view. (f): Sample Ro-58.5 (Zizyphus); rough granules of varying size may be interpreted as random structures. Scale bars: (a) = 1 mm; (b-d) = 200 µm; (e-f) = 100 µm.
